# Biological markers of lung injury before and after the institution of positive pressure ventilation in patients with acute lung injury

**DOI:** 10.1186/cc5037

**Published:** 2006-09-06

**Authors:** Magda Cepkova, Sandra Brady, Anil Sapru, Michael A Matthay, Gwynne Church

**Affiliations:** 1 The Cardiovascular Research Institute and the Departments of Medicine and Anesthesia, University of California, San Francisco, 505 Parnassus Avenue, M917, San Francisco, CA 94143-0624, USA

## Abstract

**Background:**

Several biological markers of lung injury are predictors of morbidity and mortality in patients with acute lung injury (ALI). The low tidal volume lung-protective ventilation strategy is associated with a significant decrease in plasma biomarker levels compared to the high tidal volume ventilation strategy. The primary objective of this study was to test whether the institution of lung-protective positive pressure ventilation in spontaneously ventilating patients with ALI exacerbates pre-existing lung injury by using measurements of biomarkers of lung injury before and after intubation.

**Materials and methods:**

A prospective observational cohort study was conducted in the intensive care unit of a tertiary care university hospital. Twenty-five intubated, mechanically ventilated patients with ALI were enrolled. Physiologic data and serum samples were collected within 6 hours before intubation and at two different time points within the first 24 hours after intubation to measure the concentration of interleukin (IL)-6, IL-8, intercellular adhesion molecule 1 (ICAM-1), and von Willebrand factor (vWF). The differences in biomarker levels before and after intubation were analysed using repeated measures analysis of variance and a paired *t *test with correction for multiple comparisons.

**Results:**

Before endotracheal intubation, all of the biological markers (IL-8, IL-6, ICAM-1, and vWF) were elevated in the spontaneously breathing patients with ALI. After intubation and the institution of positive pressure ventilation (tidal volume 7 to 8 ml/kg per ideal body weight), none of the biological markers was significantly increased at either an early (3 ± 2 hours) or later (21 ± 5 hours) time point. However, the levels of IL-8 were significantly decreased at the later time point (21 ± 5 hours) after intubation. During the 24-hour period after intubation, the PaO_2_/FiO_2 _(partial pressure of arterial oxygen/fraction of the inspired oxygen) ratio significantly increased and the plateau airway pressure significantly decreased.

**Conclusion:**

Levels of IL-8, IL-6, vWF, and ICAM-1 are elevated in spontaneously ventilating patients with ALI prior to endotracheal intubation. The institution of a lung-protective ventilation strategy with positive pressure ventilation does not further increase the levels of biological markers of lung injury. The results suggest that the institution of a lung-protective positive pressure ventilation strategy does not worsen the pre-existing lung injury in most patients with ALI.

## Introduction

Despite advances in intensive care, acute lung injury (ALI) is associated with a mortality of 35% to 40% and an incidence of approximately 200,000 cases per year in the U.S. [1]. Studies in Europe indicate a similarly high mortality [2]. The only therapeutic modality that has improved the survival in ALI is a lung-protective ventilation strategy [3-5].

The mechanisms by which a lung-protective ventilation strategy confers a mortality benefit are incompletely understood, but a reduction of the lung injury that leads to the release of pro-inflammatory cytokines is one likely mechanism. Structural disruption of the lung caused by mechanical ventilation (barotrauma and volutrauma) includes a component of associated mediator release (biotrauma) which can further aggravate lung injury and potentially lead to systemic multi-organ failure [6-10]. Plasma levels of interleukin (IL)-6, IL-8, surfactant protein D (SP-D), and soluble tumour necrosis factor receptor I/II (sTNFrI/II) are elevated in patients with ALI, their levels change in response to different ventilation strategies, and interestingly, this response is rapid [11-15]. Furthermore, baseline levels of IL-6, IL-8, SP-D, intercellular adhesion molecule-1 (ICAM-1), von Willebrand factor (vWF), and TNFrI/II in patients with ALI are associated with worse clinical outcomes [12-14,16-18].

However, in patients with ALI who are spontaneously ventilating with supplemental oxygen, it is not known whether the institution of positive pressure ventilation exacerbates the pre-existing lung injury. It is possible that endotracheal intubation followed by the institution of a lung-protective ventilation strategy with a lower tidal volume and a plateau pressure less than 30 cm H_2_O would not worsen already established ALI. On the other hand, it is also possible that the institution of even a lung-protective positive pressure ventilation strategy would worsen lung injury simply because the injured alveoli are exposed to some level of positive airway pressure. Stuber *et al*. [15] reported that plasma cytokine levels in patients with ALI change within 1 hour of a change in ventilation strategy. In this study, because direct assessment of extravascular lung water, lung vascular permeability, and histology is not feasible in most spontaneously ventilating patients with ALI, we measured biological markers that have been shown to change in patients with ALI with different ventilation strategies [11-15]. We reasoned that, if the institution of positive pressure ventilation increased the severity of lung injury, the levels of pro-inflammatory cytokines (IL-6 and IL-8) [14] and markers of endothelial (vWF) [18] and epithelial (ICAM-1) [19] injury would increase in the 24-hour period after the initiation of positive pressure ventilation. Therefore, we measured biomarker levels before and after endotracheal intubation. The measurements of the biochemical and physiologic indices were extended to include a full 24 hours after the institution of positive pressure ventilation.

## Materials and methods

### Study design and patient selection

A prospective observational cohort study was conducted in the intensive care unit of a tertiary care university hospital. The protocol was approved by the Institutional Committee on Human Research, and informed consent was obtained from the patients or the surrogates. All patients with ALI admitted to the adult intensive care unit of Moffit Hospital (University of California at San Francisco, CA, USA) between December 2004 and August 2005 were eligible for the study. Inclusion criteria were age of 18 years or older, positive pressure ventilation via an endotracheal tube or tracheostomy, and diagnosis of ALI/acute respiratory distress syndrome (ARDS) within 4 hours of intubation. ALI was defined according to the American-European Consensus Conference criteria: PaO_2_/FiO_2 _(partial pressure of arterial oxygen/fraction of the inspired oxygen) ratio less than 300 for ALI and less than 200 for ARDS, acute onset of bilateral infiltrates on a chest radiograph, and pulmonary artery wedge pressure less than 18 mmHg or no clinical evidence of left atrial hypertension. By definition, patients could not be diagnosed with ALI until they required intubation and the fraction of inspired oxygen was precisely known. However, most patients enrolled in the study were identified as probably having ALI before intubation, based on their tachypnea, hypoxemia, and bilateral infiltrates. The ventilation strategy of the patients was determined by their critical care physicians but was generally in concordance with the ARDS Network (ARDSNet) protocol [5], in which the tidal volume/ideal body weight is reduced toward a target of 6 ml/kg as tolerated, maintaining the plateau pressure at less than 30 cm H_2_O. The tidal volume is increased to 7 to 8 ml/kg in patients with severe dyspnea if the plateau pressure remains below 30 cm H_2_O. Patients were excluded if they had severe chronic obstructive pulmonary disease (defined as FEV1 [forced expired volume in 1 second] less than 50% predicted, a prior history of intubation secondary to chronic obstructive pulmonary disease, receiving home oxygen therapy, or chronic systemic steroids), chronic interstitial lung disease, or history of lung transplantation.

### Clinical data collection

The medical record for each patient was reviewed, and clinical data were collected using a standardised data collection form. The primary etiology of ALI was assessed based on a detailed review of the clinical history. Sepsis was defined as suspected infection and presence of at least two of the SIRS (systemic inflammatory response syndrome) criteria. Pneumonia was defined as new infiltrates on a chest radiograph and the presence of at least two of the following three criteria: fever (temperature of more than 38.3°C), leukocytosis (white blood cell count more than 12,000), or purulent secretions. Aspiration had to be witnessed or there had to be an aspiration of gastric contents from the endotracheal tube. Demographic data were recorded on day 1, and relevant physiologic data were recorded at several time points during the first 24 hours and then on days 1 and 2 after the inclusion in the study. APACHE II (acute physiology and chronic health) scores at the time of admission to the intensive care unit were calculated.

### Serum sample collection and biomarker measurements

Blood that had been obtained from routine laboratory draws was used to measure the biomarkers of lung injury. This facilitated the acquisition of pre-intubation samples while keeping sample collection and processing consistent between the pre- and post-intubation samples. Serum samples were centrifuged by the clinical laboratory at 3,000 *g *for 10 minutes at -4°C and stored at 4°C. Serum samples were retrieved from the clinical laboratory within 24 hours of collection and processed according to the research laboratory protocol. The supernatant was aspirated from the serum samples within 24 hours, aliquoted, and stored at -70°C in our research laboratory. All serum samples were assayed for IL-6, IL-8, ICAM-1, and vWF. Commercially available enzyme-linked immunosorbent assays were used to measure serum levels of IL-6 and IL-8 (Endogen [Pierce Biotechnology, Inc.], Rockford, IL, USA), ICAM-1 (Parameter; R&D Systems, Inc., Minneapolis, MN, USA), and vWF (Asserachrom; Diagnostica Stago, Asnières-sur-Seine, France). All enzyme-linked immunosorbent assay analyses were performed with strict adherence to the manufacturers' guidelines. For vWF, results are expressed as a percentage of a normal pooled plasma control reference that has been assayed against a secondary standard of the 5^th ^International Standard of vWF [20]. Pre-intubation biomarker levels were measured from a serum sample collected within a 6-hour period before intubation (mean, 4 ± 2 hours). Post-intubation biomarker levels were measured from samples collected within an 8-hour period after intubation (mean, 3 ± 2 hours) and within a 12- to 26-hour period after intubation (mean, 21 ± 5 hours).

### Statistical analysis

Data analysis was conducted using STATA 9.0 (StataCorp LP, College Station, TX, USA). The values for the cytokine concentrations for IL-6, IL-8, and ICAM-1 were not normally distributed; therefore, we carried out natural log transformation to achieve normal distribution and permit the use of parametric statistical tests. The value of concentrations of vWF was normally distributed and was not log-transformed. To evaluate the differences over time of cytokine values within each group, we used repeated measures analysis of variance and paired *t *test with Bonferroni correction for multiple post hoc comparisons as appropriate. All tests of significance were two-tailed, and a *p *value of < 0.05 was considered statistically significant.

## Results

### Baseline characteristics

The baseline demographics, clinical characteristics, and primary etiology of ALI of the 25 patients with ALI included in the study are summarised in Table 1. Sepsis was present in 40% (10/25) of the patients. The physiological variables immediately after and 24 hours after intubation are summarised in Table 2. The initial mean tidal volume was 8.2 ± 2 ml/kg, whereas 24 hours after intubation the mean tidal volume was 7.2 ± 1.8 ml/kg. The level of baseline hypoxemia pre-intubation was determined by calculating the FiO_2 _according to the American Association of Respiratory Care Guidelines [21]. The mean baseline PaO_2_/FiO_2 _ratio prior to intubation was 151 ± 101. The mean PaO_2_/FiO_2 _ratio was 136 ± 73 immediately after intubation and then significantly increased to 186 ± 63 (*p *< 0.003) at 24 hours after intubation. The peak and plateau pressure airway pressures significantly decreased in the span of 24 hours (Table 2).

### Biomarker levels in spontaneously breathing patients

All of the four biological markers (IL-8, IL-6, ICAM-1, and vWF) were elevated in the spontaneously ventilating patients with ALI within the 6-hour period prior to endotracheal intubation. The median levels of the biomarkers were all elevated several fold compared with the reference standards, the biomarker levels of a general population reported by the manufacturers of the enzyme-linked immunosorbent assays (Table 3). For IL-8, 19 patients had a value greater than the upper level of the reference standard range (16.7 pg/ml); for ICAM-1, 21 patients had a value greater than the upper level of the reference standard range (306 ng/ml); and for IL-6, nine patients had a level greater than the upper level of the reference standard (149 pg/ml) and only seven patients had a value less than the reference standard mean (43 pg/ml).

### Biomarker levels after the institution of positive pressure ventilation

**Figure 1:**
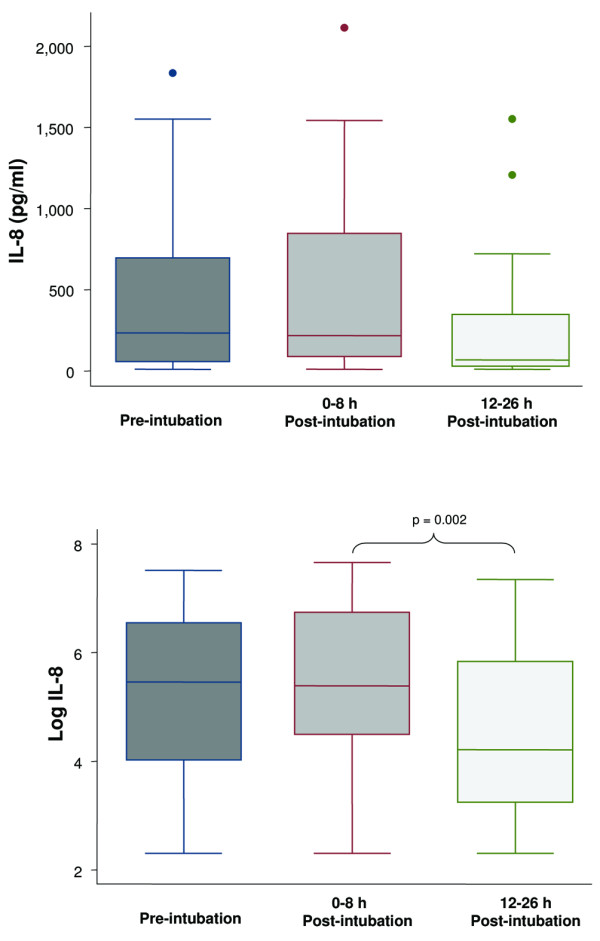
Boxplot summary of interleukin (IL)-8 levels (upper panel) and boxplot summary of log-transformed IL-8 levels to achieve normal distribution (lower panel). Median levels of IL-8 were 235 pg/ml (range, 10 to 1,836 pg/ml) pre-intubation, 219 pg/ml (range, 10 to 2,115 pg/ml) immediately post-intubation, and 68 pg/ml (range, 10 to 1,552 pg/ml) at 12 to 26 hours post-intubation. The mean levels of IL-8 after log transformation were 5.2 ± 1.8 pg/ml, 5.5 ± 1.5 pg/ml, and 4.5 ± 1.5 pg/ml, respectively. The decrease in IL-8 level at 12 to 26 hours after intubation was statistically significant (*p *= 0.002, paired *t *test with Bonferroni correction for multiple comparisons). The horizontal line represents the median, the box encompasses the 25^th ^to 75^th ^percentile, and error bars encompass the 10^th ^to 90^th ^percentile.

**Figure 2:**
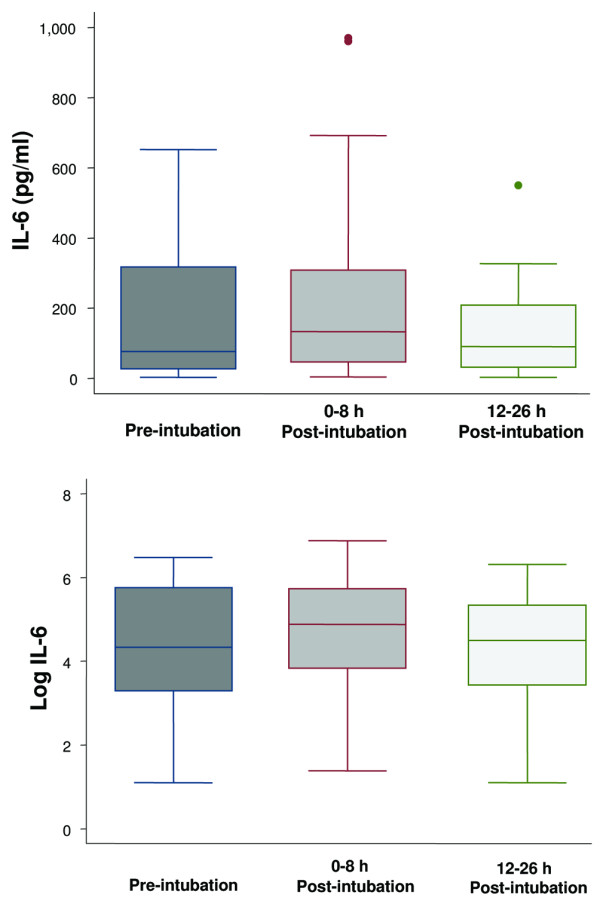
Boxplot summary of interleukin (IL)-6 levels (upper panel) and boxplot summary of log-transformed IL-6 levels to achieve normal distribution (lower panel). Median levels of IL-6 were 76 pg/ml (range, 3 to 652 pg/ml) pre-intubation, 132 pg/ml (range, 4 to 971 pg/ml) immediately post-intubation, and 90 pg/ml (range, 3 to 550 pg/ml) at 12 to 26 hours post-intubation. The mean levels of IL-6 after log transformation were 4.4 ± 1.5 pg/ml, 4.7 ± 1.4 pg/ml, and 4.2 ± 1.5 pg/ml, respectively. There was no difference among the levels of IL-6 at the three different time points (*p *= 0.34). The horizontal line represents the median, the box encompasses the 25^th ^to 75^th ^percentile, and error bars encompass the 10^th ^to 90^th ^percentile.

**Figure 3:**
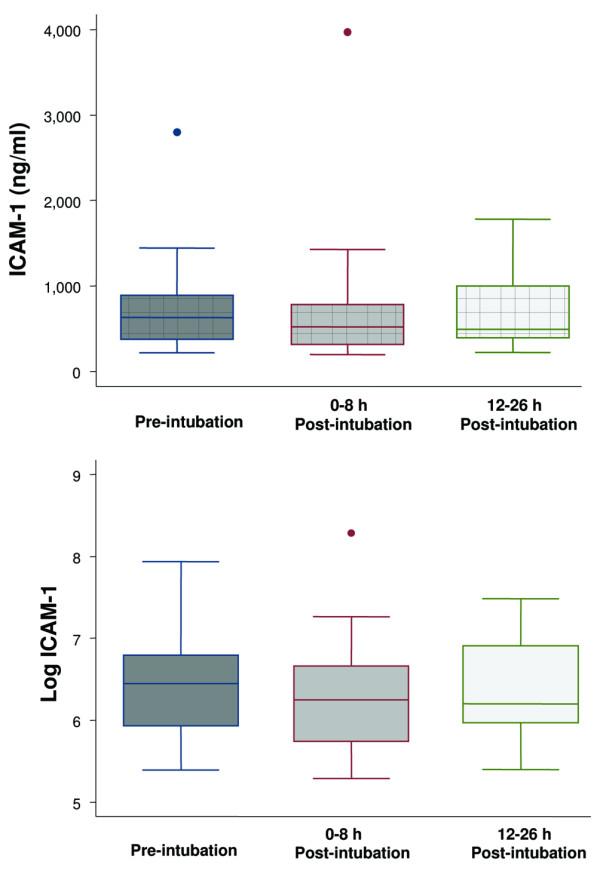
Boxplot summary of intercellular adhesion molecule-1 (ICAM-1) levels (upper panel) and boxplot summary of log-transformed ICAM-1 levels to achieve normal distribution (lower panel). Median levels of ICAM-1 were 631 ng/ml (range, 220 to 2,800 ng/ml) pre-intubation, 520 ng/ml (range, 198 to 3,970 ng/ml) immediately post-intubation, and 492 ng/ml (range, 221 to 1,780 ng/ml) at 12 to 26 hours post-intubation. The mean levels of ICAM-1 after log transformation were 6.5 ± 0.6 ng/ml, 6.3 ± 0.7 ng/ml, and 6.4 ± 0.6 ng/ml, respectively. There was no statistically significant difference among the levels of ICAM-1 at the three different time points (*p *= 0.15). The horizontal line represents the median, the box encompasses the 25^th ^to 75^th ^percentile, and error bars encompass the 10^th ^to 90^th ^percentile.

**Figure 4:**
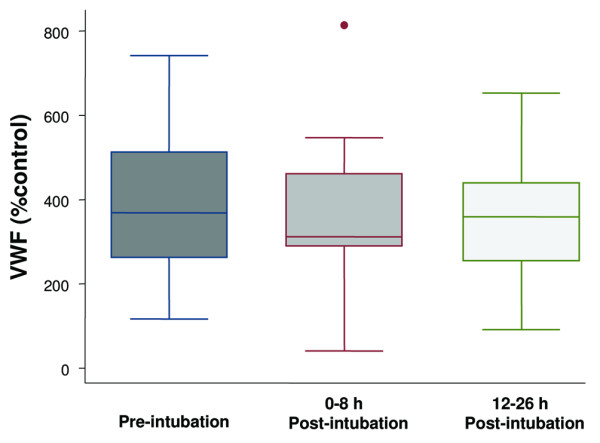
Boxplot summary of von Willebrand factor (vWF) levels expressed as a percentage of a normal pooled plasma control reference. Median levels of vWF were 368% (range, 116% to 742%) pre-intubation, 312% (range, 40% to 814%) immediately post-intubation, and 359% (range, 91% to 653%) at 12 to 26 hours post-intubation. There was no statistically significant difference among the levels of vWF at the three different time points (*p *= 0.57). The horizontal line represents the median, the box encompasses the 25^th ^to 75^th ^percentile, and error bars encompass the 10^th ^to 90^th ^percentile.

Serum cytokine levels at three different time points – within the 6 hours before intubation, within 8 hours after intubation, and between 12 and 26 hours after intubation – are shown in Table 3 and Figures 1, 2, 3, 4. The figures show boxplot summaries of actual biomarker levels and of the biomarker levels after log transformation for those biomarkers that were not normally distributed (IL-8, IL-6, and ICAM-1).

There was no statistically significant difference between the pre-intubation and immediately post-intubation levels of IL-8, IL-6, ICAM-1, or vWF. Similarly, there was no statistically significant difference between the pre-intubation levels of IL-6, ICAM-1, and vWF and the levels at 12 to 26 hours after intubation. The levels of IL-8 at 12 to 26 hours after intubation were statistically lower than the immediately post-intubation levels.

## Discussion

Previous studies of the response of biomarkers of lung injury over time in patients with ALI have focused entirely on the post-intubation phase. Biomarker levels in spontaneously ventilating patients with ALI have not been reported previously. In this study, the serum levels of IL-8, IL-6, vWF, and ICAM-1 were significantly elevated in spontaneously ventilating patients with ALI prior to the institution of positive pressure ventilation. Furthermore, our results indicate that the institution of a lung-protective ventilation strategy in patients with ALI did not significantly increase the serum levels of IL-8, IL-6, vWF, and ICAM-1.

ALI is characterised by injury to the lung endothelial and alveolar epithelial barriers, pulmonary edema, release of inflammatory mediators, and non-pulmonary organ failure. Several biomarkers of inflammation (IL-6, IL-8, and sTNFrI/II) and epithelial (SP-D) and endothelial (vWF) injury as well as adhesion molecules (ICAM-1) have been shown to be predictors of morbidity and mortality in patients with ALI, indicating that the levels of these biomarkers are affected by the severity of the lung injury [12-14,17,18]. Positive pressure mechanical ventilation imposes cyclic pressure and volume stress on the lung which can disrupt the pulmonary architecture and lead to the release of inflammatory cytokines.

In animal models, high tidal volumes can precipitate lung injury and can be associated with increased cytokine production [22-26] and extra-pulmonary organ damage [27,28]. In healthy human subjects, short-term mechanical ventilation has not been shown to be associated with cytokine release, regardless of the ventilation strategy [29,30]. However, in ventilated patients with established lung injury, the ventilation strategy has been shown to impact cytokine levels. Ranieri *et al*. [11] randomly assigned 44 patients with ARDS to conventional (11.1 ml/kg, positive end-expiratory pressure [PEEP] 6.5) and lung-protective (7.6 ml/kg, PEEP 14.8) ventilation strategies and measured bronchoalveolar lavage (BAL) and plasma biomarker levels at baseline and at 36 hours after intubation. BAL and plasma levels of sTNFrI, sTNFrII, IL-6, and tumour necrosis factor-α (TNF-α) at 36 hours were significantly lower in the low tidal volume group compared with the high tidal volume group. Based on this observation, these investigators concluded that mechanical ventilation itself can lead to an increase in cytokine levels in the lung as well as systemic circulation. Interestingly, Stuber *et al*. [15] demonstrated in patients with ALI that a higher tidal volume ventilation strategy (12 ml/kg, PEEP of 5 cm H_2_O) for only six hours was associated with a significant increase in plasma IL-6, IL-10, TNF-α, and IL-1ra compared with the initial low tidal volume strategy (6 ml/kg, PEEP of 15 cm H_2_O) and also that restoration of the low tidal volume strategy resulted in a decrease of the biomarker levels back to baseline. Observations from these small single-centre studies were confirmed and extended to a large (861 patients with ALI) multi-centre NHLBI (National Heart, Lung and Blood Institute) ARDSNet trial of two ventilation strategies [5]. Patients ventilated with a low tidal volume strategy (6 ml/kg) had a greater decrease in IL-6, IL-8, and sTNFrI/II levels and attenuated rise of SP-D over time compared with those ventilated with the high tidal volume strategy (12 ml/kg) [13,14].

Although data from these published trials provide convincing evidence that a high tidal volume ventilation strategy in patients with ALI is associated with higher mortality and higher inflammatory cytokine levels, it is not known whether a low tidal volume lung-protective strategy itself would exacerbate lung injury. This is the first clinical study to address this issue. We elected to measure several biomarkers, including IL-6, IL-8, vWF, and ICAM-1. IL-8 and IL-6 are pro-inflammatory cytokines that are elevated in patients with ALI and are predictive of clinical outcomes, and their levels are altered by different ventilation strategies. vWF is a biomarker of endothelial activation and injury, and ICAM-1 is an adhesion molecule present on epithelial and endothelial cells of the lung. Both vWF and ICAM-1 levels in patients with ALI have been shown to be associated with morbidity and mortality [17,18]. We measured levels of these biomarkers immediately before and after intubation because the study by Stuber *et al*. [15] demonstrated that the changes of inflammatory cytokine levels after modification of ventilatory strategy were very rapid (within 1 hour), but we also included another measurement (mean 21 hours after intubation) to detect changes that may occur later. In contrast to the previous studies, the levels of the biomarkers that we measured did not increase. In fact, the level of IL-8 was significantly lower at the later time point. Thus, the institution of a low tidal volume strategy in patients with ALI may not worsen lung injury in these patients. Also, there was a statistically significant improvement in several physiologic indices of lung function (Table 2), findings that correlated with more ventilator-free days in the recent ARDSNet fluid conservative therapy trial [31].

Our study has some limitations. We sampled serum but not the air spaces in these ALI patients, but BAL of the distal air spaces in non-intubated patients would not have been feasible. Furthermore, several studies have reported that plasma biomarkers change in response to changes in ventilation strategies, indicating that BAL samples may not be necessary to the interpretation of changes in cytokine levels. The initial ventilation strategy differed among the patients, because this was not a controlled trial. The immediately post-intubation mean tidal volume of 8 ml/kg probably reflects a delay in diagnosis of ALI/ARDS and the subsequent implementation of the ARDSNet protocol. The tidal volume of 7.2 ml/kg ideal body weight at 24 hours after intubation is consistent with the effort to decrease the tidal volume to 6 ml/kg. It is possible that, if the ventilation strategies had been in greater concordance with the ARDSNet protocol earlier on, our results may have been different; however, it is unlikely that it would change our acceptance of the null hypothesis, namely that the institution of positive pressure ventilation is not associated with an increase in biomarkers of lung injury. The total number of patients in this study (*n *= 25) was modest but, for two reasons, was sufficient to rule out a significant increase in the biological markers of inflammation or endothelial and epithelial injury after the institution of positive pressure ventilation. There were actually a statistically significant decrease in IL-8 levels and a trend toward a decrease in all biomarker levels at 12 to 26 hours (Figures 1, 2, 3, 4). The differences in the levels of IL-6, ICAM-1, and vWF at the three different time points were so minimal that it is not likely that more patients would have shown a completely different response than we observed. Also, the oxygenation data and plateau airway pressures showed an improvement in lung function that was statistically significant even in this modest number of patients.

## Conclusion

Inflammatory cytokines and biological markers of endothelial and epithelial injury are elevated in spontaneously ventilating patients with ALI, and the institution of a lung-protective positive pressure ventilation strategy does not increase these levels. This suggests that a lung-protective ventilation strategy does not exacerbate pre-existing lung injury in most patients with ALI.

## Key messages

• Biological markers of lung injury are elevated in spontaneously ventilating patients with ALI.

• Intubation and institution of a lung-protective positive pressure ventilation strategy does not further increase the levels of biological markers of lung injury.

## Abbreviations

ALI = acute lung injury; ARDS = acute respiratory distress syndrome; ARDSNet = ARDS Network; BAL = bronchoalveolar lavage; FiO_2 _= fraction of the inspired oxygen; ICAM-1 = intercellular adhesion molecule-1; IL = interleukin; PaO_2 _= partial pressure of arterial oxygen; PEEP = positive end-expiratory pressure; SP-D = surfactant protein D; sTNFrI/II = soluble tumour necrosis factor receptor I/II; TNF-α = tumour necrosis factor-α; vWF = von Willebrand factor.

## Competing interests

The authors declare that they have no competing interests.

## Authors' contributions

GC and MAM designed the study. GC performed data acquisition. GC and SB performed the immunoassays. MC and MAM performed the data analysis and interpretation and drafted the manuscript. MC and AS performed the statistical analysis. All authors read and approved the final manuscript.

## Figures and Tables

**Table 1:** Clinical characteristics of 25 patients with acute lung injury or acute respiratory distress syndrome

Clinical characteristic	No. of patients (percentage of total)^a^
Age	62 ± 21 years^b^
Males	13 (52)
APACHE II score	27 ± 9^b^
	
Primary etiology of ALI/ARDS	
Pneumonia	16 (64)
Sepsis	4 (16)
Aspiration	4 (16)
Other	1 (4)
	
Underlying medical illness	
Chronic liver disease	6 (24)
Chronic renal insufficiency	6 (24)
Metastatic cancer	0 (0)
Hematologic malignancy	3 (12)
AIDS	1 (4)
Diabetes mellitus	1 (4)

^a^Except where marked with superscript b; ^b^data shown as means ± standard deviation. ALI, acute lung injury; APACHE II, acute physiology and chronic health evaluation; ARDS, acute respiratory distress syndrome.

**Table 2:** Physiologic variables immediately after intubation and 24 hours after intubation

Physiologic variables	Within 1 to 2 hours after intubation^a^	Twenty-four hours after intubation^a^	*p *value^b^
PaO_2_/FiO_2 _ratio	132 ± 71	186 ± 63	0.003
Plateau pressure (cm H_2_O)	26 ± 8	22 ± 4	0.02
Peak inspiratory pressure (cm H_2_O)	32 ± 7	27 ± 5	0.02

Positive end-expiratory pressure	6.2 ± 3	7.2 ± 3	0.16
Oxygenation index	12 ± 2	9 ± 2	0.21
Quasistatic respiratory compliance (ml/H_2_O)	27 ± 12	29 ± 10	0.32
Mean airway pressure (cm H_2_O)	12 ± 3	12 ± 4	0.53
Tidal volume (ml)	480 ± 120	409 ± 80	0.01
Tidal volume per kg IBW (ml/kg)	8.2 ± 2.2	7.2 ± 1.8	0.02

^a^Data shown as mean ± standard deviation; ^b^paired *t *test comparing the pre-intubation and 24-hour post-intubation variables. FiO_2_, fraction of inspired oxygen; IBW, ideal body weight; PaO_2_, partial pressure of arterial oxygen.

**Table 3:** Biomarker levels pre-intubation and at two time points post-intubation

Biological marker	Reference standard, mean (range)	Pre-intubation, median (range)	0 to 8 hours post-intubation, median (range)	12 to 26 hours post-intubation, median (range)	*p *value^a^
IL-6 (pg/ml)	43 (0 to 149)	76 (3 to 652)	132 (4 to 971)	90 (3 to 550)	0.34
IL-8 (pg/ml)	9 (1.2 to 16.7)	235 (10 to 1,836)	219 (10 to 2,115)	68 (10 to 1,552)	0.0003^b^
ICAM-1 (ng/ml)	211 (115 to 306)	631 (220 to 2,800)	520 (198 to 3,970)	492 (221 to 1,780)	0.15
vWF	% control	368 (116 to 742)	312 (40 to 814)	359 (91 to 653)	0.58

^a^Repeated measures analysis of variance comparing levels of cytokine at three different time points; ^b^see Figure 2. ICAM-1, intercellular adhesion molecule-1; IL, interleukin; vWF, von Willebrand factor.
